# Collective knowledge: using a consensus conference approach to develop recommendations for physical activity and nutrition programs for persons with type 2 diabetes

**DOI:** 10.3389/fendo.2012.00161

**Published:** 2012-12-11

**Authors:** Tanya R. Berry, Catherine B. Chan, Rhonda C. Bell, Jessica Walker

**Affiliations:** ^1^Faculty of Physical Education and Recreation, University of AlbertaEdmonton, AB, Canada; ^2^Department of Agricultural, Food and Nutritional Science, Faculty of Agriculture, Life and Environmental Sciences, University of AlbertaEdmonton, AB, Canada; ^3^Department of Physiology, Faculty of Medicine and Dentistry, University of AlbertaEdmonton, AB, Canada

**Keywords:** diabetes, lifestyle interventions, consensus conference, physical activity, nutrition

## Abstract

The purpose of this consensus conference was to have a lay panel of persons with type 2 diabetes (T2D) work in collaboration with an expert panel of diabetes professionals to develop strategies designed to improve dietary and physical activity adherence in persons with T2D. Lay panel participants were 15 people living with T2D. The seven experts had expertise in exercise management, cardiovascular risk factors, community-based lifestyle interventions, healthy weight strategies, the glycemic index, exercise motivation, and social, environmental and cultural interactions. All meetings were facilitated by a professional, neutral facilitator. During the conference each expert gave a 15-min presentation answering questions developed by the lay panel and all panel members worked to generate suggestions for programs and ways in which the needs of persons with T2D may be better met. A subgroup of the lay panel used the suggestions created from the conference to generate a final list of recommendations. Recommendations were categorized into (1) diagnosis/awareness (e.g., increasing awareness about T2D in the general public, need for lifelong self-monitoring post-diagnosis); (2) education for the person with diabetes (e.g., periodic “refresher” courses), professionals (e.g., regular interactions between researchers and persons with T2D so researchers better understand the needs of the affected population), and the community (e.g., support for families and employers); and (3) ongoing support (e.g., peer support groups). The recommendations from the conference can be used by researchers to design and evaluate physical activity and nutrition programs. The results can also be of use to policy makers and health promoters interested in increasing adherence to physical activity and nutrition guidelines among persons with T2D.

## INTRODUCTION

The prevalence of diabetes in Canada is increasing, doubling within the Canadian province of Alberta between 1997 and 2007 to a rate of 4.5% (Johnson and Balko, 2009). Adults with diabetes are much more likely to need health care than people without type 2 diabetes (T2D) costing, in total, three to four times that of people without T2D (Johnson et al., 2009). Thus, finding effective treatments for this disease are essential. Healthy self-management practices such as following a recommended diet, regular physical activity, appropriate medication use, self-monitoring of blood glucose, and smoking cessation (Canadian Diabetes Association Clinical Practice Guidelines Expert Committee, 2008), can be successful (Minet et al., 2010) and are cost-effective from a payer perspective over 10 years (The Diabetes Prevention Program Research Group, 2012). Thus, increasing adherence to lifestyle interventions can benefit both the client and the health care system.

The clinical practice guidelines from the Canadian Diabetes Association Clinical Practice Guidelines Expert Committee (2008) provide physical activity and nutrition behavior recommendations. However, they do not address how the recommendations may be achieved. As a consequence, uptake of recommended lifestyle behaviors appears to be modest at best (Plotnikoff et al., 2006; Resnick et al., 2006). Although popular views suggest that diet and physical activity choices are primarily controlled by the individual, the role of interpersonal and environmental factors in the dietary and physical activity choices people ultimately make are increasingly recognized (Johnson and Balko, 2009).

Diabetes educators identified complex and interwoven reasons for lack of client understanding and uptake of lifestyle recommendations (Berry et al., 2012). It was felt that capacity of clients to change was influenced by social, environmental, cultural, and personal factors. Tension between educators and their clients was also noted; educators felt that clients sometimes perceived the educators as the “diabetes police.” In addition, this research identified a lack of physical activity expertise in the Albertan diabetes education setting, as most educators come from nursing or dietitian backgrounds. Less is known regarding what people with T2D consider to be the most effective approach to lifestyle education. A meta-synthesis of focus groups conducted across eight European countries found that patients with T2D were confused by conflicting information, felt stigmatized because of their disease, and had difficult relationships with health care providers who blamed them for their disease (Vermeire et al., 2007). It was also perceived that health care providers had little credibility because they don’t know what it’s like to have the disease. These findings suggest that intervention development for dietary and physical activity adherence must include both client and expert perspectives.

One way to incorporate multiple perspectives in the development of interventions is to use consensus conference methods. Consensus conferences allow for the provision of “the views of informed citizens and their key issues of concern” (People and participation, n.d.) which allows for knowledge exchange around key issues (The Loka Institute, n.d.) including diabetes management (Royal College of Physicians of Edinburgh, 2010). A focal purpose of a consensus conference is the altering of a power balance between the priority given to factual expertise and lay perspectives in relation to a particular topic (Nielsen et al., 2006). A defining feature is the exchange and consensual development of knowledge using experts and a panel of lay citizens. By involving diabetes experts and persons with T2D in a conference dialog focused on physical activity and nutrition intervention for T2D, the consensual development of intervention recommendations can be achieved. This may aid in addressing some of the issues raised by diabetes educators (Berry et al., 2012) and persons with T2D (Vermeire et al., 2007).

Although it can be argued that having T2D makes one an “expert” of experiences of the disease, it is also likely that many persons with T2D lack disease-related medical and scientific information. Therefore, for the purposes of this consensus conference, participants with T2D were considered as members of the “lay panel” whereas researchers and physicians with expertise in T2D were considered members of the “expert panel.” The purposes of the consensus conference were: (1) to involve persons with T2D and experts in the consensual development of intervention recommendations which participants believe will optimize adherence to physical activity and nutrition guidelines; and (2) to provide a forum for knowledge dissemination around existing strategies related to physical activity and nutrition intervention for T2D through expert presentations to a varied audience, including the T2D clients, diabetes educators, and the general public.

## CONSENSUS CONFERENCE PROCEDURES

All aspects of the research were approved by a university research ethics board and all participants provided written informed consent prior to participating in the conference.

### PARTICIPANTS

#### Lay panel

Fifteen individuals (six men and nine women) were recruited from existing databases of persons with T2D who had indicated interest in participation in research and projects. In accordance with consensus conference procedures, participants were selected because they were confident and autonomous and thus able to participate in an interactive forum (Nielsen et al., 2006). They were not necessarily representative of the Canadian T2D population, but did represent a range of time since diagnosis (less than a year to more than 30 years). All participants were older than 40 years. No other demographic information was collected. It was important to purposefully select people who could contribute to the overall aim of the conference (i.e., to develop an intervention). When first contacted, potential participants were told of the purpose and structure of the conference, including their role as members of the lay panel and the intent of the preparatory day and conference proceedings. Participants were informed that through group nomination on the preparatory day, a subgroup would be selected to develop final recommendations, as recommended for consensus conferences. Participants received a $100 honorarium and travel costs were reimbursed.

#### Expert panel

Seven T2D experts were identified by members of the research team. They represented a range of diabetes specializations including exercise management, cardiovascular risk factors, community-based lifestyle interventions, healthy weight strategies, glycemic index, social, environmental and cultural interactions, and exercise motivation. All were active researchers with PhD degrees and two also had medical degrees and were practicing physicians in addition to being researchers. Panel members received a $100 honorarium and travel costs were covered.

The process followed consensus conference guidelines and occurred in three stages: a lay panel planning meeting, the conference, and a lay panel final meeting (Nielsen et al., 2006).

### PLANNING MEETING

The lay panel met with the moderator 1 month before the conference to develop a question for each of the seven diabetes experts. Participants also chose eight individuals amongst themselves to comprise a lay panel subgroup whose task was to develop the final recommendations after the conference. A professional facilitator moderated all meetings to ensure a fair and independent process unbiased by a research agenda.

Participants were told the purpose of the conference was to generate recommendations for interventions that they believe would be appropriate and feasible for others with T2D in Alberta. Participants were introduced to the consensus conference approach, including the structure and goal of the conference. Research team members gave two presentations on current best practices in nutrition and physical activity interventions for T2D and the strengths and weaknesses of current education programs and interventions. Following the presentations, the members of the research team left. With the support of the facilitator, lay panel participants developed specific questions for each of the experts. Immediately following the planning meeting, the questions were given to members of the expert panel so they could prepare their presentations in advance of the conference.

The seven questions developed were:

1. How do external factors, such as genetically modified foods, food additives, medications, and pesticides, contribute to obesity and diabetes susceptibility?
2. Tell us trusted internet sources that have proven to be the most effective with behavior change in weight management for all income levels.
3. What do you have to do to keep physical activity and nutritional interventions effective and adaptable in the long-term?
4. Can you recommend exercise or fitness programs that incorporate a consideration of the Alberta climate and a range of physical and financial limitations of participants?
5. Please explain the workings of the glycemic index and how to use this information to create an effective diet.
6. How do we promote awareness of diabetics and current issues to shape positive attitudes of the community and families of diabetics
7. What are the key behaviors required to produce compliance to regular exercise programs and what kinds of things provide motivation, combat depression and develop the self-discipline to sustain long-term diabetic health?

### CONSENSUS CONFERENCE

The conference day had two sessions. In the morning, each of the experts gave a presentation answering the question developed for him or her by the lay panel. This session was public and diabetes educators, health promotion specialists, and the public were invited. Approximately 50 people attended including members of the research team. The facilitator moderated the session and following each presentation there was a question and answer period.

In the afternoon, only members of the lay and expert panels and the facilitator were present. During this session the facilitator moderated discussion by the expert and lay panel members as they generated ideas and suggestions about T2D interventions.

### LAY PANEL MEETING

The day following the consensus conference, the lay panel subgroup, in a meeting moderated by the facilitator, produced a document outlining a final set of recommended strategies and intervention elements.

### RECOMMENDATIONS

The results presented are those developed by the lay panel, not the researchers. Three overarching themes were identified: diagnosis, education, and support. Participants also advocated that “as a group we have to be more vocal.” Rather than express feelings of victimization, participants acknowledged they needed to take an active part in disease management and believed the conference was a valuable step in that process. Participants also recommended that the word “program” replace “intervention” because the word intervention is potentially misleading for the lay person and associated with detrimental behaviors such as drug use and smoking. They also suggested the development of programs not only for diabetics (their preferred term) but also for researchers who may have specific disease knowledge but lack experience of living with the disease and thus also in need of education.

### DIAGNOSIS

The panel suggested that diagnosis begins with increased disease awareness among the general public and within the diabetic community and that lifelong self-monitoring starts immediately following diagnosis. Specific recommendations made ranged from mass media campaigns to increase awareness of where to access information about T2D to free community blood sugar testing clinics. Another key suggestion was to have better first points of contact (e.g., with pharmacists or physicians) on where to get local support and education.

### EDUCATION

Education recommendations included suggestions for the person with diabetes, for researchers, and for the community.

The panel proposed periodic refresher courses to provide progressive and updated diabetes education. The current method of providing information and counseling when first diagnosed was considered ineffective. The lay panel advocated for ongoing behavioral counseling to aid with overcoming barriers to exercise (e.g., time management), managing appetite, and similar issues. There is also a need for diabetes specific exercise programs. Finally, the psychological effects of diabetes, such as depression, needs to be part of education. Furthermore, some co-morbid conditions require medication and help is needed on how to manage diabetes in conjunction with co-morbidities and associated treatment. Thus, the recommendations encompass ongoing education across the behavioral and psychological aspects of diabetes.

There should be more face to face time between those with diabetes and researchers to allow for information exchange. Researchers may learn about what diabetics believe are the pressing issues that need to be addressed. Diabetics can learn about the latest research findings. As written in the summary statement “it’s a two way street – researchers/stakeholders will know what the public wants and the public will know what the researchers/stakeholders are doing.” The consensus conference was given as an example of such a meeting. It was also recommended that physicians need better training and ongoing education on diet and exercise. In particular, it was perceived that family physicians were not prepared to provide behavioral counseling.

The community is also in need of ongoing education. The panel defined community members as family, employers, and general community members. Businesses (e.g., food stores, restaurants) with diabetic clients should be educated regarding the specialized needs of diabetics. One suggestion was that adult continuing education classes could be provided. Even if such classes already exist, there is a lack of awareness of them among persons with diabetes. Within the Alberta context it was reported that many online resources were out of date.

### SUPPORT

The theme of support overlapped somewhat with the themes of diagnosis and education, but the panel believed it to be distinct. One of the key comments was “you are your own support”; that is, diabetics should be encouraged to take responsibility for their own support (e.g., by joining a peer support group). The idea of ongoing education was reiterated but additional ideas were included such as greater media attention. The panel pointed out that members of the media were invited to the conference but none showed up. They believed this highlighted the problem of promoting discussion and raising awareness around T2D. The lay panel reported learning new information by participating in the conference (e.g., the importance of checking blood sugar after exercise was new information for the majority of participants) but it is a challenge to get this information to the general public. “One stop shopping” is needed to optimize access to information and training.

## DISCUSSION

This conference provided a forum for persons with T2D and experts to develop consensual recommendations designed to optimize adherence to physical activity and nutrition guidelines. The need for multilevel interventions was confirmed. Although the final recommendations made encompass the role of the individual in taking some responsibility for their own health, community and societal level contributors to the disease were also identified. The results affirm the “complex and interwoven” factors cited by diabetes educators as contributors to difficulties in changing diet and physical activity behaviors (Berry et al., 2012). However, the consensus conference panel participants did not criticize diabetes educators. Rather, they advocated for continuing education for patients, practitioners, researchers, and the public. The conclusions of a meta-analysis highlight education as a useful technique in diabetes self-management (Minet et al., 2010). However, Minet et al. (2010) also concluded that the most effective way to effect change in an intervention has yet to be determined.

Many of the program recommendations made during this consensus conference echo the results of a meta-synthesis of European focus groups. For example, people feel they lack information regarding progression of T2D and need ongoing information rather than just education when initially diagnosed (Vermeire et al., 2007). These authors also stress that information is often given at inappropriate times or is difficult to understand or remember, a point also raised by the diabetes educators in Berry et al.’s2012 study. Where the consensus conference results diverge from the findings of Vermeire et al. (2007), is in the role of health services. Vermeire et al. (2007) state that in a European context at least, adherence to lifestyle behaviors is dependent on individual beliefs and attitudes and their relationship with health care providers, rather than with health care systems in general. However, a diabetes consensus conference conducted in the UK in 2010 concluded that individuals, family, communities, governments, and the health system are all needed to prevent and treat T2D (Royal College of Physicians of Edinburgh, 2010. We concur with this viewpoint. Although participants in the Alberta consensus conference argued for patient responsibility they also recognized multilevel problems that make adherence to lifestyle behaviors a challenge. Psychological and educational programs are also included in the list of recommendations in the UK conference statement. The Royal College of Physicians of Edinburgh (2010) concluded that it is not just patients who need education, but “all those involved in the care of patients should be informed by applied psychology and include the need for a person-centered approach.” Thus, our Canadian results substantiate those in other countries. It is also important to note that the Canadian conference recommendations were made by non-scientific “lay persons.” Their recommendations align with the recommendations from the scientific community and we suggest there is consensus that multilevel programs are needed that support persons with T2D when trying to change their diet and activity behaviors. Without support from the community, health services, and society, changing behavior is very difficult. As Goyder et al. (2010) pointed out, understanding the determinants of behaviors at a population level can help shift behavior at an individual level.

In summary, this conference gave persons living with T2D a chance to express what they believe will improve attempts to change lifestyle behaviors relative to T2D and the overall well-being of diabetics. It was recommended that researchers and health care professionals continue to engage keen members of the general public who have T2D in regular meetings to discuss ideas and present possible solutions to improve the quality of life for those living with diabetes. This is an important suggestion that is unique to this conference. Others (e.g., Baranowski, 2006) have pointed out that how to effect successful behavior change is still unknown. It may be that through ongoing dialog between all stakeholders more effective programs can be developed. It has also been argued that a “paradigm shift” is needed in the prevention of diabetes. This would require prioritizing the dissemination of prevention studies and ensuring the cost-effectiveness of prevention programs (Cefalu, 2012). The cost-effectiveness issue is important. Although the panel did not make any specific recommendations regarding access to education across socioeconomic groups, they did advocate for free education when possible. This is an important consideration given those with the lowest income are more likely to have diabetes (Rabi et al., 2006).

This conference allowed professionals and citizens to discuss relevant topics surrounding T2D together, hear each other’s point of view, and create possible solutions together. Participants spoke of their excitement and interest in future changes to the health care system as a result of their recommendations. They were passionate about the topic chosen, the procedure of the conference and enthusiastic about the overall results. If these recommendations are effectively implemented, they may help to increase adherence to physical activity and nutrition guidelines to ensure a long and healthy life for all Canadians.

## Conflict of Interest Statement

The authors declare that the research was conducted in the absence of any commercial or financial relationships that could be construed as a potential conflict of interest.
